# Protecting providers and patients: results of an Internet survey of health care workers’ risk perceptions and ethical concerns during the COVID-19 pandemic

**DOI:** 10.1186/s12245-021-00341-0

**Published:** 2021-03-24

**Authors:** Lauren O’Neal, Michele Heisler, Ranit Mishori, Rohini J. Haar

**Affiliations:** 1 School of Public Health, University of California Berkeley, Berkeley, CA USA; 2 Physicians for Human Rights, New York, NY USA; 3 Department of Internal Medicine, University of Michigan School of Medicine and School of Public Health, Ann Arbor, MI USA; 4 Department of Family Medicine, Georgetown University School of Medicine, Washington, DC USA; 5 Division of Epidemiology and Biostatistics, School of Public Health, University of California Berkeley, Berkeley, CA USA

**Keywords:** Coronavirus, COVID-19, Pandemic, Health care workers, Risk perception, Ethics, Resource allocation, Prioritization, Scarcity

## Abstract

**Background:**

The COVID-19 pandemic has generated worldwide scarcity of critical resources to protect against and treat disease. Shortages of face masks and other protective equipment place health workers, already on the frontline of the disease, at higher risk. Moral distress from making difficult decisions about allocating scarce resources and care to patients ill with COVID-19 can further add to burdens health workers face. This study investigates clinical health workers’ risk perceptions and concerns about the ethics of their clinical decision-making, the actions of their institutions to address resource scarcity concerns during the COVID-19 pandemic, and their ability to voice safety concerns, as well as their own views on how scarce resources should be allocated.

**Methods:**

An online survey was open to health care workers who provide clinical care to patients, with no specialty training or geographic location requirements, from May 19 to June 30, 2020. Participants were recruited through purposive sampling using medical association and institutional email lists, and by snowball sampling.

**Results:**

Of 839 participants, a majority were physicians (540, 69.4%) working in academic medical centers (270, 35.2%) or private health systems in the community (234, 30.5%) in the USA (760, 90.7%). Most reported being concerned about their own health (494, 73.6%) and about the possibility of spreading COVID-19 to family and friends (534, 85.9%) during the pandemic. All respondents reported shortages or rationing of at least one type of medical resource (e.g., sanitizing supplies and personal protective equipment). More than half of respondents (351, 53.9%) did not feel they received sufficient training in how to allocate scarce resources in the pandemic. Many felt moral distress related to conflicts between institutional constraints and what they believed was right (459, 66.5%). Though a majority (459, 67.7%) reported feeling “comfortable” internally communicating with their administration about safety issues, far fewer reported feeling “confident” speaking publicly about safety issues without retaliation from their institution (255, 37.3%).

**Conclusions:**

In the face of limited resources, surveyed health care workers reported concern about their own and their families’ health from exposure. Securing adequate protective equipment must be a high priority for pandemic management. In addition, more governmental and facility-level ethical guidance is required for allocation of resources given ongoing scarcity, and facilities must create conditions so health care workers can speak openly about safety issues without fear of retaliation.

**Supplementary Information:**

The online version contains supplementary material available at 10.1186/s12245-021-00341-0.

## Introduction

In December 2019, the first cases of Coronavirus Disease 2019 (COVID-19) were reported in Wuhan, China. The disease was initially characterized by severe respiratory symptoms caused by a highly infectious, novel, human-infecting coronavirus [1]. The virus has spread rapidly since its emergence, and on March 11, 2020, the World Health Organization declared a COVID-19 pandemic [2]. By mid-October, over 37 million cases of COVID-19 had been confirmed globally, with over one million deaths [3]. While much remains to be learned about the characteristics of COVID-19, it is a highly infectious multi-system disease [4, 5] spread by both respiratory droplets and aerosol [6] and can cause significant mortality and morbidity [7].

Health care workers (HCWs) are at a higher risk of being exposed to infectious diseases, both during the current pandemic and during prior health crises. During prior epidemics, such as the 2003 outbreak of Severe Acute Respiratory Syndrome (SARS), health care workers reported anxiety over contracting the disease at work, infecting family members, and stigmatization by their communities [8]. This reportedly undermined public health efforts, with health care workers reporting both a reluctance to care for patients and a loss of trust in the health care system. During the current pandemic, health care workers remain a particularly high-risk group. In March, over 3300 health care workers in China were infected with the virus [9]. The same month, as many as 20% of health care workers in Italy caring for patients with COVID-19, were infected [10]. Recent studies have illustrated the effectiveness of personal protective equipment (PPE) such as face masks [11]. Shortages across the globe, however, have exacerbated already existing occupational health hazards, raising concern that poor planning and resource allocation have increased risks for health care workers [12]. Front-line health care workers were found to have at least a threefold risk of a positive COVID-19 test in comparison to the general population [13], and more than 1000 health care workers in the USA have died of COVID-19 as of late August 2020 [14].

In addition to managing their own risks, health workers are often also tasked with making decisions about scarce resources for their patients. The stress from personal risks in this setting may be compounded by moral distress, or distress caused by feeling that the “ethically correct action to take is different from what one is tasked with doing”, [15] resulting from clinical decision-making in a resource-constrained environment. In addition to PPE shortages, medical facilities have experienced and continue to experience shortages of prescription medications [16] and essential equipment, like ventilators [12, 17]. Without sufficient supplies, health care workers may have to navigate moral distress related to institutional resource allocation guidelines or lack thereof, and how it may differ from decisions they would personally make. There have been reports that health care workers in resource-constrained settings also face the possibility of retaliation if they choose to speak out about safety concerns or allocation decisions [18].

We aim to characterize the scope and specifics of this possible moral distress, elicit discussions around ethical resource allocation, and examine health care workers’ risk perceptions during the COVID-19 pandemic. In particular, we examined (i) perceptions of risk faced by HCWs and their family, (ii) experiences with protective equipment and other resource shortages. (iii) associations between reported shortages and risk perception, (iv) experiences with decision-making and resource allocation, and (v) ability to address workplace concerns.

Though the challenges faced by health care workers in the COVID-19 pandemic are not inherently new, relevant previous studies on mental health and burnout [19–22], occupational risks [23], and ethical decision-making [24, 25] are limited in this context because they were not all being experienced simultaneously, as was the case with the unprecedented nature of this global crisis. The synergism of all these issues is unique. Additionally, these issues are now also being faced in relatively high-resource health care settings that had previously not experienced such challenges, or at least not to that degree. For example, research on ethical decision-making is often carried out in humanitarian or low-resource settings; however, the pandemic has made it essential to now look at this issue in all settings where COVID-19 makes it relevant. Further exploration of these issues within the context of the pandemic will help facilitate a more robust public health response for current and future health crises. This effort can form the basis of policies to protect the health of health care workers as well as the development of ethical resource allocation strategies.

## Methods

### Study design

Investigators designed a one-time, web-based online survey with questions about health care workers’ experiences during the COVID-19 pandemic. The survey included 26 questions including questions on: (i) demographic information, like age, gender, and profession; (ii) risk perception when working with COVID-19 patients, including worry about spreading illness to family or friends; (iii) experiences making decisions about limited resources and related ethical concerns; and (iv) comfort expressing concerns when providing care to COVID-19 patients (See survey in Supplementary Materials). The final survey was designed and developed by collaborators at UC Berkeley and Physicians for Human Rights to gather a wide range of information on HCWs’ experiences during the pandemic, while remaining easy and minimally time-consuming to complete. Given the novel experiences of PPE availability and ethical resource allocation in traditionally resource rich settings in the COVID-19 pandemic, validated surveys for this purpose are not available.

### Data collection

Because the COVID-19 pandemic impacts a variety of HCWs around the world, the survey was open to all health care workers who provide clinical care to patients, including physicians, nurses, paramedics, respiratory therapists, and medical assistants. All participants providing clinical care were included regardless of geographic location, medical specialty, or specific role. We used purposive sampling through electronic listservs for health care workers (e.g., professional medical and nursing associations, international human rights and health non-governmental organizations, public health organizations). Additionally, snowball sampling was utilized by encouraging participants to forward the survey link to other health care workers in their professional and personal networks. There were no monetary or other incentives to complete the survey. Though our efforts were primarily US-based, we intentionally allowed for the inclusion of non-US perspectives because of the inherently global nature of a pandemic. We selected this sampling strategy to maximize the number of participants in our survey and collect a diversity of experiences. Given the rapidly changing context of the pandemic, we believe being able to reach as many HCWs as quickly as possible warranted some of the limitations of this strategy, which will be discussed further. Survey data was collected using Qualtrics (licensed by the University of California, Berkeley) from May 19 to June 30, 2020.

### Data analysis

Data was collated and cleaned prior to analysis, which was conducted with data from respondents who provided consent and answered at least one demographic question (*n* = 839). Because respondents were not required to answer every question, analyses of specific survey questions only consider completed responses and remove blank or null responses; as a result, respondent count varies from question to question. Descriptive statistics were reported on aggregated data. Chi-squared testing was used to test possible correlates related to demographics, region, or specialty but was limited secondary to significant heterogeneity and quality limitations that would preclude extensive disaggregated data analysis. Data cleaning and analysis were completed using R (v 4.0.2), RStudio (v 1.3.1056), and Excel (v 16.41).

This research was approved and exempted by the Institutional Review Board of the University of California, Berkeley (Protocol # 2020-03-13152).

## Results

Over a 6-week survey period from May 19 to June 30, 2020, we received 938 individual entries into the online survey, of which 839 individuals (89.4%) completed questions beyond the initial screening questions. We report response frequencies and percentages (*n*, %) for each question because not all respondents answered all survey questions.

The mean age of participants was 46.8 years (range 19–92). The majority were female (66.7%), physicians (69.4%), and had an average of 16.9 years in clinical practice (range 0–66). A majority of respondents provided care in academic medical centers (35.2%) or private health systems in the community (30.5%). Demographic data is summarized in Table 1.

**Table 1:** Characteristics of survey participants

***Gender***	***Count***	***Percent***
Female	557	66.7
Male	273	32.7
Other	5	0.6
Total	835	100.0
***Profession***		
Critical Care Registered Nurse/Nurse Anesthetist	5	0.6
Doctor (MD, DO)	540	69.4
Laboratory technician	3	0.4
Licensed practical nurse	1	0.1
Nurse practitioner	31	4.0
Paramedic or EMT	10	1.3
Physician assistant	11	1.4
Mental Healthcare Provider	52	6.7
Registered nurse	74	9.5
Respiratory therapist	2	0.3
Other	49	6.3
Total	778	100.0
***Facility type***		
Academic medical center	270	35.2
Community, private health system	234	30.5
Government Health System (i.e., county or city hospital)	113	14.7
Long-term care or assisted living	7	0.9
Out of hospital, ambulance etc.	9	1.2
Prison or other detention health system	4	0.5
VA health system	16	2.1
Other	114	14.9
Total	767	100.0
***Relevant prior experiences***		
Yes	249	32.3%
*Disasters or emergencies in home country*	*57*	*7.4*
*Global Health development work*	*78*	*10.1*
*Military*	*15*	*1.9*
No	522	67.7
Total	771	100.0

**Fig. 1:**
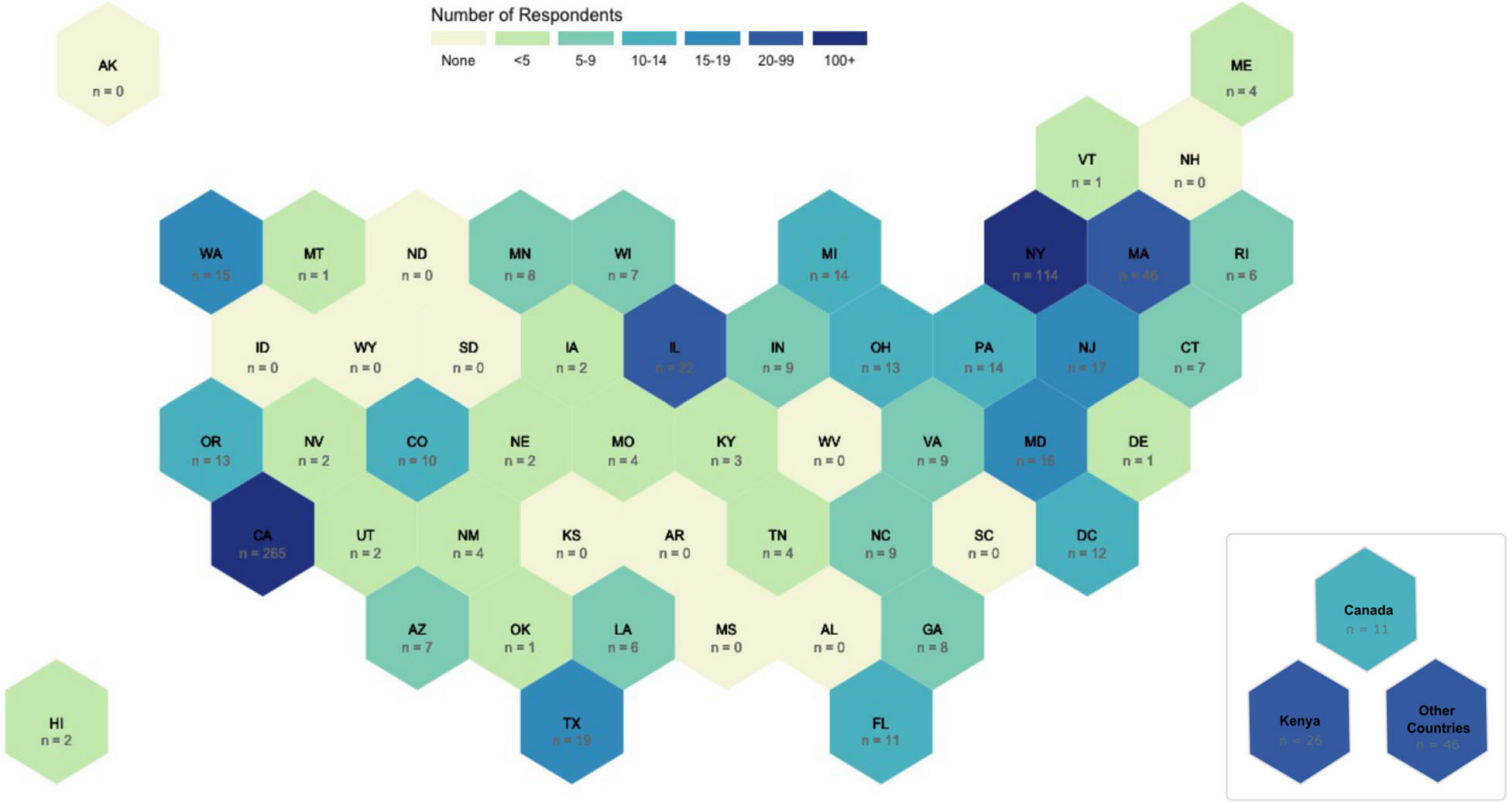
Location of respondents (including US states)

Among the 27 countries in which respondents reported working, 760 (90.7%) worked in the USA, 26 (3.1%) worked in Kenya, 11 (1.3%) worked in Canada, and 41 (4.9%) were in other countries (see Supplementary Material). Though the majority of respondents work in the USA, snowball sampling led to the inclusion of responses gathered participants from around the world. While the geographic location of loci of responses is not representative, it does highlight the global scope of the COVID-19 pandemic. Of 711 health care workers practicing in the USA who reported a state, 265 (37.3%) worked in California, 114 (16.0%) in New York, and 46 (6.5%) in Massachusetts (Fig. 1).

### Risk perception

Participants were asked about personal and professional circumstances that may influence their perception of risk during the COVID-19 pandemic. Most respondents delivered clinical care (in-person or telemedicine) for patients with or suspected of having COVID-19 (584, 76.4%) and reported a lower than usual patient volume for that time of year (432, 75.1%). Additionally, 523 (68.1%) respondents did not have personal health risk factors and 485 (63.2%) did not have members of their household with risk factors for COVID-19. Personal risk factors included an underlying medical condition, immunocompromised status, or pregnancy, while household member risk was defined as living with a person who is elderly, with a chronic health condition, or with another high-risk factor for complications from COVID-19 infection.

Health care workers were concerned about their own health (494, 73.6%) and about the possibility of spreading COVID-19 to family and friends (534, 85.9%) during the pandemic. Significantly more health care workers with at least one personal health risk factor reported worrying about their personal health (86.5%) than those without personal health risk factors (68.2%) (*p* < 0.05, Table S2). Similarly, a significantly greater proportion of health care workers living with a person with health risk factors reported worrying about spreading COVID-19 (89.7%) than those without household risk factors (84.0%) (*p* < 0.05, Table S3).

#### Perceived risks and PPE availability

Of 733 respondents who provided information about PPE in their place of work, 461 (62.9%) reported PPE shortages. Across the three countries with most respondents, the USA, Kenya, and Canada, the proportion of health care workers reporting PPE shortages was similar (63.1%, 63.6%, 60% respectively). Of the 29 respondents from other countries who provided information about PPE, 17 (58.6%) reported shortages. High proportions of respondents from the US states with the highest survey participation, California, New York, and Massachusetts, also reported PPE shortages (60.6%, 66.7%, 76.9%, respectively). Of the 271 participants from all other US states, 168 (62.0%) also reported PPE shortages.

**Fig. 2:**
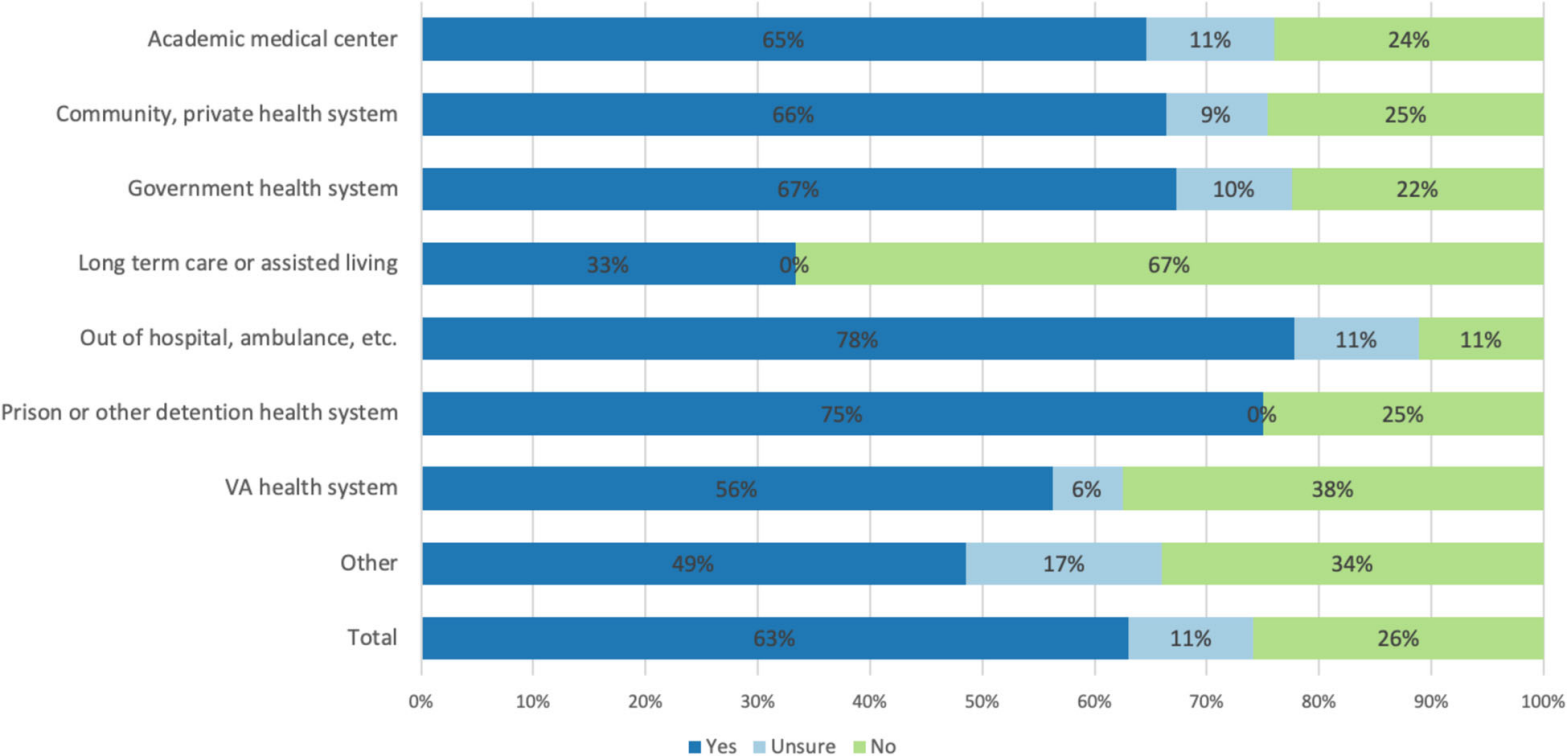
Reports of PPE shortages by health care setting. Survey Question: “My place of work has faced or is facing shortages of personal equipment (PPE)”

Health care workers worked in a variety of care delivery settings, all of which experienced PPE shortages. A majority of health care workers reported a shortage of PPE at their workplace in all settings except long-term care facilities and those in the “other” category which respondents could describe in open text (e.g., retired, federally qualified health care center, private practice) (Fig. 2). There was no significant difference between the rates of PPE shortages reported by physicians (333, 65.0%) and HCWs who are not physicians (128, 58.2%) (*p* > 0.05).

**Fig. 3:**
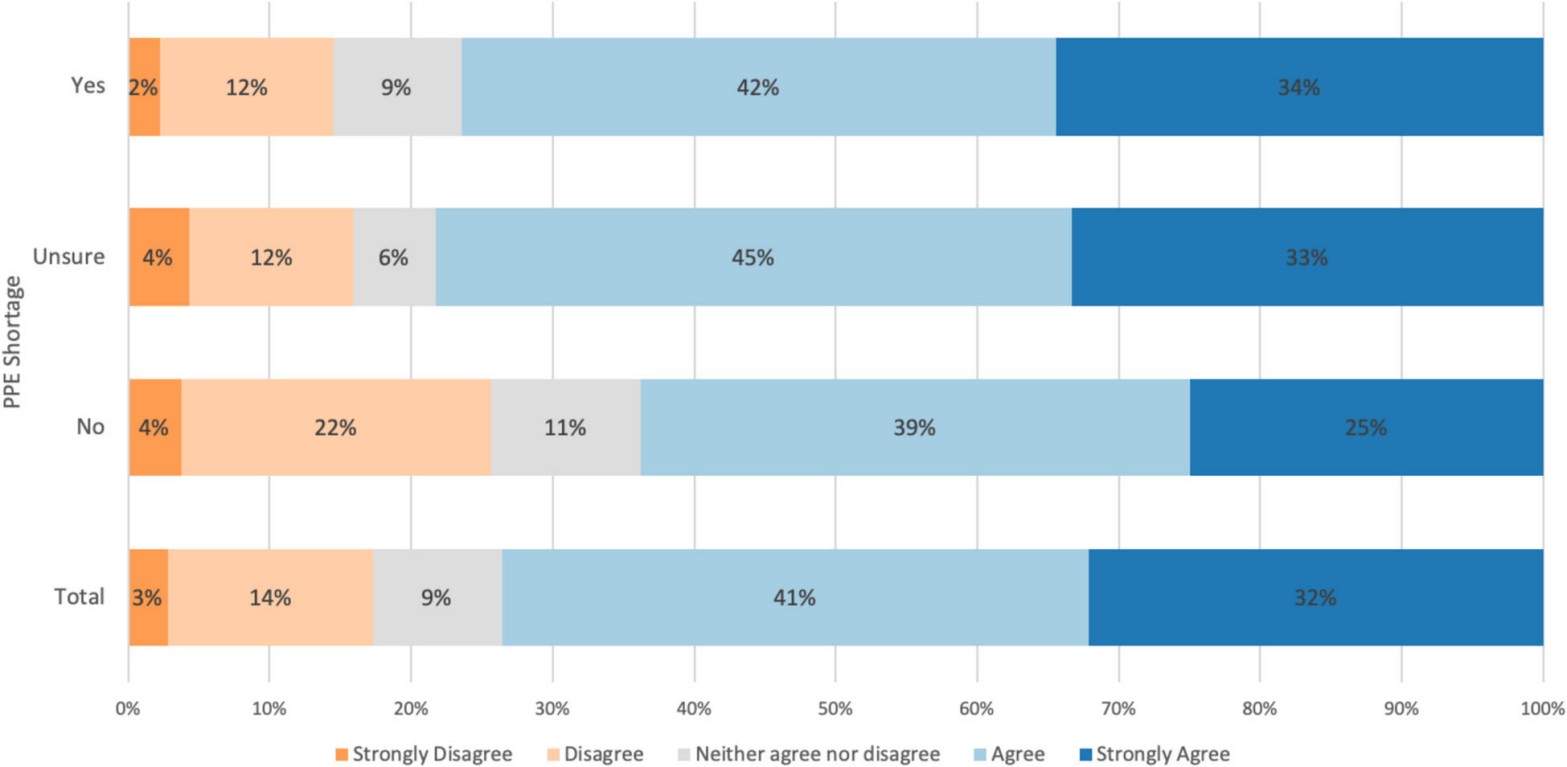
Impact of PPE shortages on personal health worry. Survey questions. Personal health worry: “I feel worried about my personal health if/when providing direct in-person care to COVID-19 patients. ”PPE shortage: “My place of work has faced or is facing shortages of personal equipment (PPE)”

Health care workers’ concern about their own health was impacted by PPE shortages in their workplace (Fig. 3). A greater proportion of respondents who worked in facilities that had PPE shortages were worried about their personal health (337, 76.4%) than those who did not report facility PPE shortages (156, 68.1%). There was a statistically significant relationship between reported PPE shortages and health care worker worry about personal health (*p* < 0.05). By contrast, there was no significant difference in respondents who believed it was their duty to provide in-person care to COVID-19 patients if adequate PPE was unavailable between facilities with PPE shortages (179, 33.0%) and without (73, 30.4%) (Figure S1).

### Ethical decision-making and resource allocation

In addition to reported high rates of PPE shortages across health care facilities and geographic locations, all respondents experienced rationing or limited availability of a variety of supplies and equipment. All health care workers who responded to a question about limiting or rationing resources (*n* = 544) reported limited availability of at least one of the listed critical medical supplies or equipment (Table 2).

**Table 2:** Reported limiting or rationing of medical resources

***Survey question:*** ***“My facility or office is actively limiting/rationing (Select all that apply):”***	***Count***	***Percent***
Personal protective equipment (PPE)	470	86.4
Disinfectants, sanitizers and other cleaning supplies	319	58.6
Diagnostic testing (such as COVID-19 tests or antibody tests)	291	53.5
Ventilators/respirators	34	6.3
Other modes of assisted ventilation (such as BiPAP and CPAP)	72	13.2
Other therapeutic equipment	49	9.0
Hospital beds	44	8.1
None of the above	0	0

Though similar proportions of health care workers had training or experience with priority setting with limited resources (44.3% agree or strongly agree, 44% disagree or strongly disagree), a majority of respondents disagreed (53.9%) when asked if they had received sufficient training or preparation in how to allocate scare resources during the COVID-19 pandemic (Figure S2). There was no significant difference in training for resource allocation decisions between professions (physicians and non-physicians) and between health care facility types. Despite insufficient training, a slightly greater proportion of health care workers did not feel worried about personally making decisions regarding resource allocation (46.48% disagree or strongly disagree, 34.22% agree or strongly agree). In contrast, more than half of health care worker participants were concerned that their belief in what is right would conflict with institutional constraints or procedures when allocating limited resources (17.4% with current concerns, 49.1% worried about future conflict). This documented moral distress reflects the weight of ethical concerns HCWs must grapple with when making decisions about how to allocate limited resources.

The survey also probed health care workers regarding their preferences for how to ration scarce life-saving resources. Table 3 displays HCWs’ rankings, with each row showing the proportion of respondents who selected a prioritization strategy for a particular ranked choice (e.g., first choice, second choice). When presented with different possible prioritization strategies, most respondents favored prioritizing patients who would be most likely to survive based on their clinical picture, with this strategy making up 46.3% of the first-choice rankings and 25.4% of the second-choice rankings. This prioritization was followed by strategies that focus on young patients with a greater potential to live a longer life (often known as the fair innings model), and health care workers. A majority (63.49%) placed a strategy that prioritizes people of political, economic, or cultural importance as one of the last two options.

**Table 3:** Ranking of prioritization strategies for ethical decision-making

***Prioritization strategy***								
*Ranking*	Survival^a^	Youth^b^	HCWs^c^	Sickest^d^	First In^e^	Lottery^f^	VIP^g^	Other^h^
1	46.3%	10.3%	22.6%	11.1%	4.0%	2.4%	0.5%	2.9%
2	25.4%	31.2%	16.1%	14.0%	7.6%	3.2%	1.3%	1.3%
3	13.8%	26.7%	27.3%	14.1%	8.1%	7.2%	1.7%	1.0%
4	5.9%	14.6%	15.1%	23.2%	18.3%	15.1%	7.0%	0.8%
5	4.9%	8.6%	8.1%	18.4%	27.8%	22.3%	8.7%	1.1%
6	1.7%	6.2%	7.6%	11.3%	24.2%	30.0%	17.2%	1.7%
7	1.6%	2.4%	2.9%	6.7%	9.2%	17.5%	51.0%	8.7%
8	0.3%	0.0%	0.3%	1.1%	0.8%	2.4%	12.6%	82.5%
Average Ranking	2.1	3.1	3.1	3.9	4.7	5.2	6.4	7.5

Survey question: “Imagine you were required to make your institution’s policy on how to ration scarce life-saving resources to patients. Of the following SEVEN approaches, please rank in order how you might prioritize patients. Please mark 1 for your first choice, 2 for your second choice, 3 for your third choice, etc.”

^a^Prioritize people who are most likely to survive based on clinical picture regardless of other factors

^b^Prioritize young people who have greater potential to live a longer life

^c^Prioritize health care workers

^d^Prioritize the sickest people regardless of other factors

^e^Use a “First Come, First Served” approach until equipment runs out

^f^Use a lottery system to give everyone a fair shot

^g^Prioritize people who are important political, business, or cultural figures

^h^Another prioritization approach

### Addressing workplace concerns

**Fig. 4:**
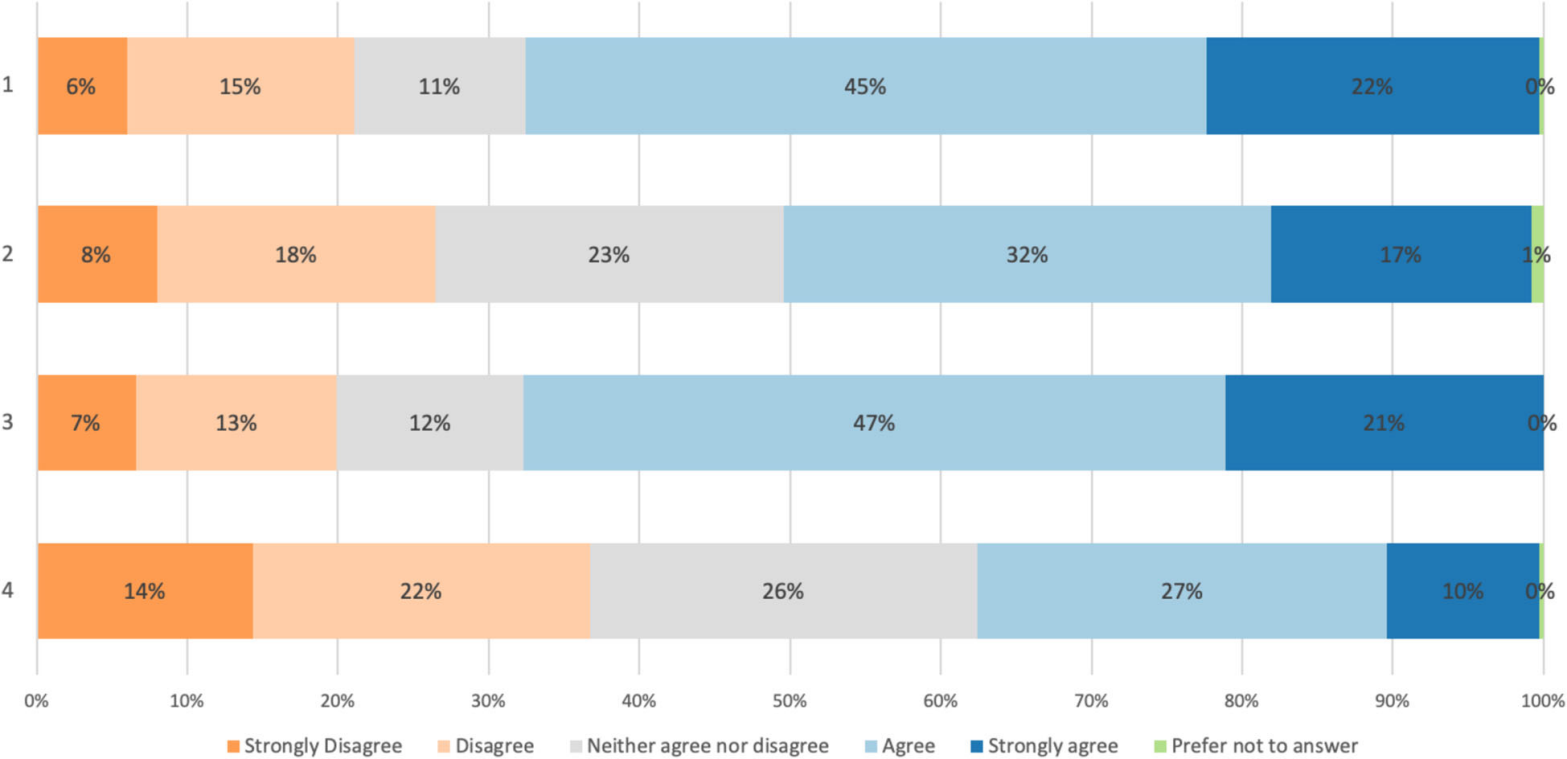
HCW experiences addressing safety concerns. Survey Questions. 1: “I am willing to do tasks outside my own formal training to care for critically ill patients with COVID-19.” 2: “If my hospital or clinic runs out of adequate PPE, I will be unwilling to provide in-person to patients with COVID-19.” 3: “I feel comfortable communicating with administration about safety issues in my institution related to the care of COVID-19 patients.” 4: “I feel confident that if I spoke out publicly about safety issues in my institution related to the care of COVID-19 patients, I would not experience retaliation from my institution”

Health care workers reported concerns about their own health and the health of their social circles due to COVID-19 exposure, PPE and medical equipment shortages, and a lack of training to allocate scarce resources. These reports warrant further investigation of how health care workers feel about the work they are asked to do during the COVID-19 crisis and if they can speak about these issues in the workplace and beyond (Fig. 4).

When asked if they would complete tasks outside of their own formal training to care for patients with COVID-19 (e.g., a surgeon being assigned to an internal medicine COVID-19 unit), a majority of respondents agreed that they would be willing to do so (67.3% agree or strongly agree, 21.1% disagree or strongly disagree). This willingness to do tasks outside of formal training did not extend to situations in which health care workers did not have PPE; almost half of respondents (49.6%) were unwilling to provide in-person care to patients with COVID-19 if their workplace ran out of adequate PPE.

Given reported PPE shortages and established concerns about providing in-person care to COVID-19, it is important to understand how health care workers feel they can communicate about these issues. Though a majority of respondents felt comfortable communicating internally with their administration about safety issues (67.7%), a smaller proportion would feel confident speaking publicly about safety issues without retaliation from their institution (37.3%). There were no significant differences between genders or professions (physicians or non-physicians) with respect to communication about safety issues internally or externally (*p* > 0.05).

## Discussion

The public health crisis created by the COVID-19 pandemic has resulted in critically important questions about scarce health care resources and ethical decision-making. As cases of COVID-19 continue to surge in the fall and winter of 2020, there are reports of limited resources across the USA and the world. In this setting, lessons on ethical resource allocation are timely and may inform how this pandemic, and future ones, are managed. In this survey of health care workers providing direct clinical care across the US states, several countries, and a variety of health care facility types, a large majority of health care workers reported feeling worried about their own health and the health of their family and friends related to their provision of care to COVID-19-positive patients. In combination with reported PPE and equipment shortages across sectors, health care workers experienced moral distress from conflict between what they believe is right and institutional constraints or procedures when allocating limited resources. They reported insufficient training to handle the issues of scarcity rampant during the pandemic. Given the established health care worker stress related to risk perception and ethical decision-making, this research also investigated how health care workers addressed these concerns. Though many health care workers felt confident bringing safety issues into discussion with their administration internally, they did not feel as comfortable discussing these concerns publicly without retaliation from their workplace.

This study contributes to prior research on HCW experiences during the COVID-19 pandemic in several key ways. By examining health care workers’ risk perception, we add to the numerous studies investigating HCW knowledge of or experience with COVID-19 [26–29] and the psychological impacts of the pandemic [30–37]. Despite the pandemic’s documented negative psychological impacts on HCWs and significant resource limitations, fewer studies have characterized the relationship between HCWs’ perceptions of COVID-19, workplace safety and resource availability, and administrative responsiveness to crisis [38, 39]. Our work expands on these by demonstrating HCWs’ moral distress due to resource limitations and discomfort conveying concerns about workplace safety or policies. It is important to understand the context of these negative outcomes in order to mitigate or even prevent them. To our knowledge, we are also the first survey of HCWs to investigate how respondents would personally prefer to allocate scarce resources, which provides crucial insight to reducing future moral distress.

The information gathered during this pandemic is critical to the future of an industry already struggling with burnout [20–22] and personnel shortages [40–42]. We have shown that this crisis exacerbates the challenges that currently hamper the quality of health care for patients and those working within the field. If we do not use this as a stress test to understand the areas for improvement within our health care infrastructure, we have wasted an opportunity to make lasting change for the better. The documented moral distress and risk perceptions of HCWs point to clear avenues for future action: improved training and clear conversations about priority setting. Beyond this, our research makes a unique contribution by delving into the contexts surrounding HCWs’ distress. By outlining the factors surrounding the negative impacts of the pandemic, including administrative support and guidelines for scarce resource allocation, we have shown concrete areas for change.

### Limitations

This study had a number of significant limitations. As an Internet survey, sampling was not random but rather was through investigators’ personal networks, non-governmental organizations and other professional associations' listservs, and snowball sampling. Thus, respondents may not be representative of the entire health care worker community. Disaggregating the data by country, region or other demographic factors would thus not be suitable and may be misinterpreted. While the survey included participants from a wide range of health care professions, countries, and care facility types, a majority were physicians practicing in academic medical centers and/or private health systems in California, New York, and Massachusetts. Thus, the results of this analysis are limited in their ability to represent the experiences of health care workers outside of these groups and regions. Future surveys or research may consider stratifying sampling by region, country, or profession to better understand how these factors play a role in the experience and opinions of respondents. In addition, as in all survey research, the meaning of some of the responses was open to interpretation by the respondents in their unique contexts. Moreover, the severity and scope of the COVID-19 pandemic is constantly changing over time and across world regions. The design of the survey as a single point-in-time data collection strengthened its ability to represent real-time attitudes and experiences but limited its ability to capture to changes over time. However, the aim of this study was to understand the range of issues heath care workers experience while providing care in a pandemic and contribute to the ongoing process of enhancing the safety of providers and patients when planning for pandemic events. We believe that this large-scale effort provides considerable insight into health care worker risk perception and ethical decision-making.

## Conclusions

This survey, while not representative of all health care workers, highlights the experiences of almost 900 clinicians who reported concerns about their own safety, the safety of their families, and the health of their patients. Shortages of essential medical resources on health workers and patients resulted in moral distress among these respondents and can lead to severe stress for the health workers for their families and their patients. Though improved preparation would ideally limit the necessity of rationing of resources, we have shown that it is critical to [1] provide health care workers with sufficient training and support to make decisions in situations of resource scarcity and [2] to consider health care workers’ ethical priorities when shaping institutional resource allocation policies to reduce moral distress. Federal or state level guidance on ethical prioritization of scare resources that incorporates feedback from HCWs on how they believe resources should be prioritized could significantly alleviate the moral distress that individual clinicians can face when making difficult resource allocation decisions. Health care facilities should also consider how to support employee communication to enhance workplace safety. Though increased preparation to meet increased medical resource needs would improve health care worker safety, policies that increase health care workers confidence in communication about safety issues without retaliation will be a critical step to reducing workplace risks for health care workers. Future research on what state or federal rationing policies currently exist or should be developed, as well as on deeper exploration of various rationing strategies and risks to the physical and emotional well-being of health care workers in the setting of the COVID-19 pandemic, will be important next steps. The COVID-19 pandemic may be the first modern situation where considering these issues from a global perspective is both possible and necessary, but it certainly will not be the last.

## Supplementary Information

**Additional file 1: Table S1.** Countries Where Respondents Work. **Table S2.** Personal Health Concerns between HCWs with and without Health Risk Fasctors. **Table S3.** Disease Spread Concerns between HCWs with and without Household Risk Factors. **Figure S1.** Impact of PPE Shortages on HCW Duty to Provide Care. **Figure S2.** HCW Experiences with Resource Limitations and Allocation

**Additional file 2.** COVID-19 HCW Questionnaire

## Data Availability

The datasets generated and analyzed during the current study are not publicly available due to the Institutional Review Board approved protocol, but all code for analysis is available from the corresponding author on reasonable request.
