# Supplementary material for: Protecting providers and patients: results of an Internet survey of health care workers’ risk perceptions and ethical concerns during the COVID-19 pandemic
Source: Int J Emerg Med. 2021 Mar 24;14:18. doi: 10.1186/s12245-021-00341-0 (PMC7988645; doi:10.1186/s12245-021-00341-0)
Supplement: Supplementary file 1 — Additional file 1: Table S1. Countries Where Respondents Work. Table S2. Personal Health Concerns between HCWs with and without Health Risk Fasctors. Table S3. Disease Spread Concerns between HCWs with and without Household Risk Factors. Figure S1. Impact of PPE Shortages on HCW Duty to Provide Care. Figure S2. HCW Experiences with Resource Limitations and Allocation [file 12245_2021_341_MOESM1_ESM.docx]

**Supplementary Material**

Table S1: Countries Where Respondents Work

| **Country** | **Count** | **Percent** |
| --- | --- | --- |
| Australia | 1 | 0.12% |
| Bangladesh | 1 | 0.12% |
| Bolivia | 1 | 0.12% |
| Canada | 11 | 1.31% |
| China | 1 | 0.12% |
| Egypt | 1 | 0.12% |
| France | 2 | 0.24% |
| Georgia | 1 | 0.12% |
| Germany | 1 | 0.12% |
| India | 1 | 0.12% |
| Israel | 3 | 0.36% |
| Kenya | 26 | 3.10% |
| Kuwait | 1 | 0.12% |
| Lebanon | 2 | 0.24% |
| Netherlands | 2 | 0.24% |
| Nigeria | 2 | 0.24% |
| Portugal | 1 | 0.12% |
| Saudi Arabia | 1 | 0.12% |
| Serbia | 1 | 0.12% |
| South Africa | 1 | 0.12% |
| Sweden | 1 | 0.12% |
| Syria (Opposition Control) | 1 | 0.12% |
| Tunisia | 1 | 0.12% |
| Turkey | 9 | 1.07% |
| United Kingdom of Great Britain and Northern Ireland | 4 | 0.48% |
| United States of America | 760 | 90.69% |
| Zimbabwe | 1 | 0.12% |
| **Grand Total** | **838** | **100.00%** |

Table S2: Personal Health Concerns between HCWs with and without Health Risk Factors

|  | Some Risk Factors | No Risk Factors | Row Total |
| --- | --- | --- | --- |
| Worried about Personal Health^a^ | 173 (86.5%) | 321 (68.2%) | 694 |
| Not Worried about Personal Health^b^ | 27 (13.5%) | 150 (31.8%) | 177 |
| Column Total | 200 (29.8%) | 471 (70.2%) | 671 |

Survey Question: “I feel worried about my personal health if/when providing direct in-person care to COVID-19 patients.”

^a^Includes “Strongly Agree” and “Agree” responses

^b^Includes “Neither agree nor disagree”, “Disagree”, and “Strongly Disagree” responses

Table S3: Disease Spread Concerns between HCWs with and without Household Risk Factors

|  | Some Risk Factors | No Risk Factors | Row Total |
| --- | --- | --- | --- |
| Worried about Spread^a^ | 200 (89.7%) | 335 (84.0%) | 535 |
| Not Worried about Spread^b^ | 23 (10.3%) | 64 (16.0%) | 87 |
| Column Total | 223 (35.9%) | 399 (64.1%) | 622 |

Survey Question: “After providing direct in-person care to COVID-19 patients I am worried about spreading COVID-19 to my family or friends.”

^a^Includes “Strongly Agree” and “Agree” responses

^b^Includes “Neither agree nor disagree”, “Disagree”, and “Strongly Disagree” responses

Figure S1: Impact of PPE Shortages on HCW Duty to Provide Care


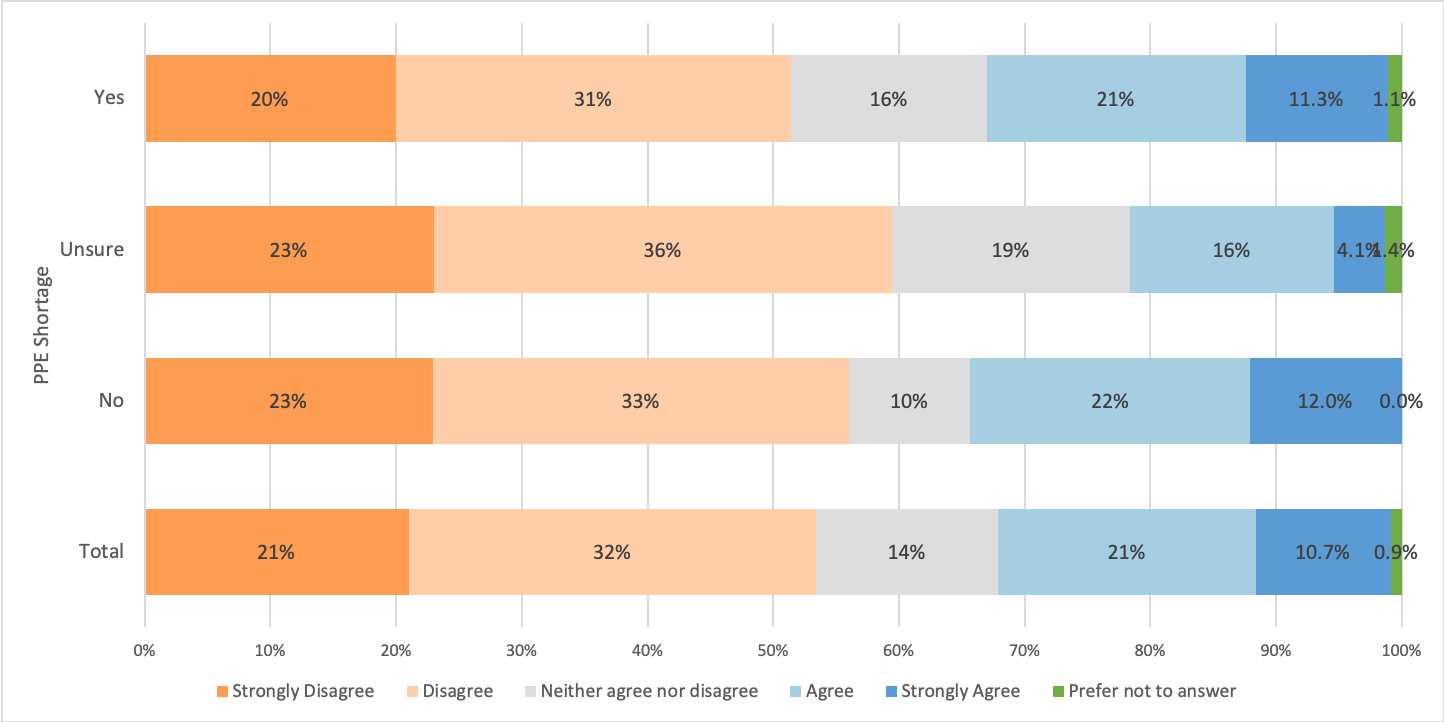


Survey Questions

Duty to Provide Care: “It is my professional duty to provide in-person care to patients with COVID-19 even if I cannot be provided with adequate personal protective equipment (PPE).”

PPE Shortage: “My place of work has faced or is facing shortages of personal equipment (PPE).”

Figure S2: HCW Experiences with Resource Limitations and Allocation


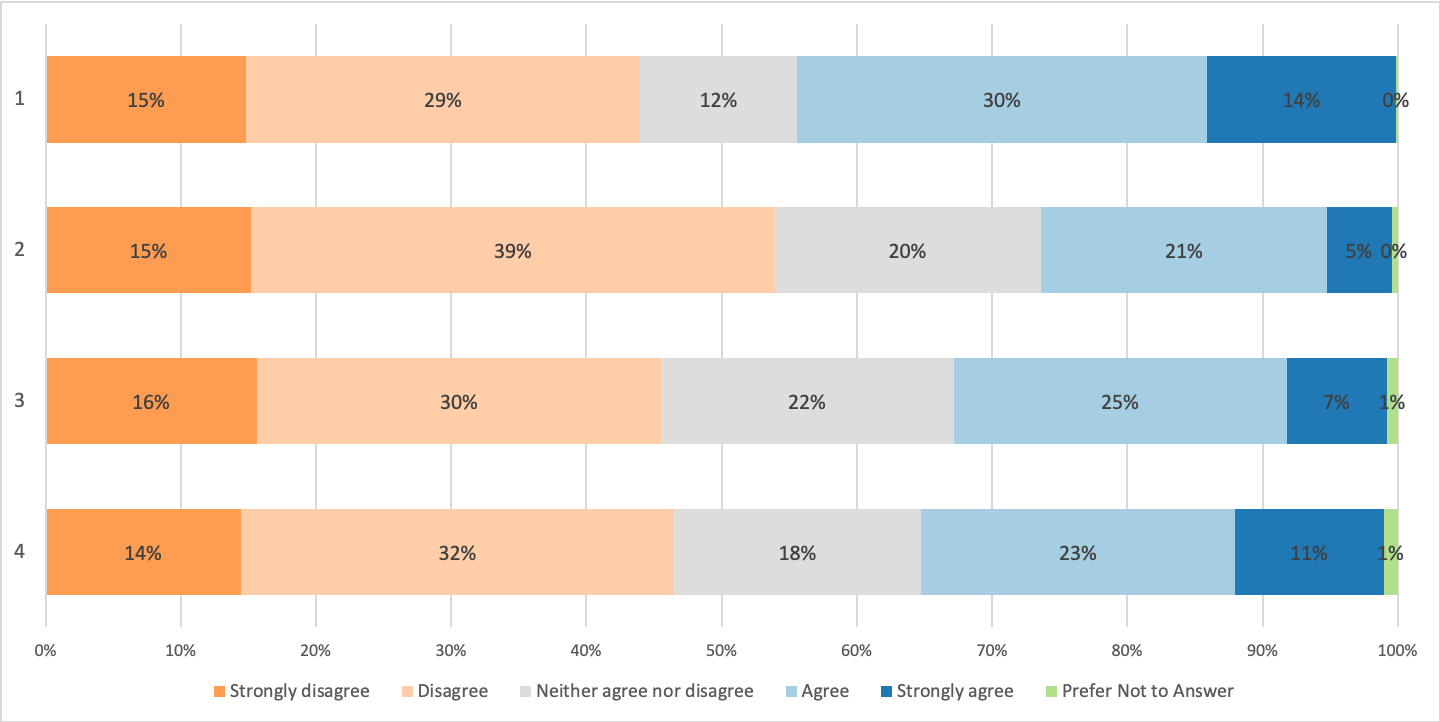


Survey Questions

1: “Before COVID-19, I was trained and/or had significant experience with priority setting with limited resources.”

2: “I have received sufficient training and preparation in how to allocate scarce resources to patients amidst the current COVID-19 pandemic.”

3: “My health care facility has given me clear information about how scarce resources will be allocated here if necessary so the burden of making decisions will not fall on the bedside team.”

4: “I am worried I will be required to personally make decisions about allocating limited resources, like which patients get ventilators or other life-saving resources, in the moment based on my own judgment.”
