# Supplementary material for: Protecting providers and patients: results of an Internet survey of health care workers’ risk perceptions and ethical concerns during the COVID-19 pandemic
Source: Int J Emerg Med. 2021 Mar 24;14:18. doi: 10.1186/s12245-021-00341-0 (PMC7988645; doi:10.1186/s12245-021-00341-0)
Supplement: Supplementary file 2 — Additional file 2. COVID-19 HCW Questionnaire [file 12245_2021_341_MOESM2_ESM.docx]

COVID-19 HCW Questionnaire

Start of Block: SECTION 1: CONSENT

Q1.1
SECTION 1: CONSENT
 
The purpose of the study is to examine healthcare worker perceptions on personal risks and protection and ethical decision making with regard to personal safety, duty to treat, and resource allocation during the COVID-19 pandemic. This voluntary study should take less than 7 minutes. The findings of this anonymous study may be published in a report and/or journal article, but we will not use your name or any other identifying information. Your responses to the survey indicate your consent for this purpose. Your responses may help inform ethical decision making and resource allocation, both during the current COVID-19 pandemic and in the future. Participation is completely voluntary. You may stop or cancel at any time. The results of this survey will not be linked in any way with your contact information.

Q1.2
Do you agree to participate AND confirm that you are a healthcare worker?

- Yes (1)
- No (2)

Skip To: End of Survey If Do you agree to participate AND confirm that you are a healthcare worker? = No

| Page Break |  |
| --- | --- |

End of Block: SECTION 1: CONSENT

Start of Block: SECTION 2: Demographic Questions

Q2.1 SECTION 2: DEMOGRAPHIC QUESTIONS

| 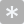 |
| --- |

Q2.2
What is your age?

________________________________________________________________

Q2.3 What is your gender?

- Male (11)
- Female (12)
- Other: (13) ________________________________________________

| 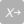 |
| --- |

Q2.4 In which country do you currently work?

▼ United States of America (187) ... Zimbabwe (1357)

Display This Question:

If List of Countries = United States of America

Q2.5 In which state do you currently work?

▼ Alabama (1) ... I do not reside in the United States (53)

| 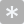 |
| --- |

Q2.6 In which city or town do you currently work?

________________________________________________________________

| 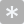 |
| --- |

Q2.7 How many years have you been in clinical practice (i.e. after school)?

________________________________________________________________

Q2.8 What is your profession?

- Doctor (MD, DO) (4)
- Critical Care Registered Nurse/Nurse Anesthetist (6)
- Registered Nurse (7)
- Nurse Practitioner (8)
- Physician Assistant (9)
- Licensed Practical Nurse (13)
- Paramedic or EMT (14)
- Respiratory Therapist (16)
- Laboratory Technician (17)
- Psychologist, clinical social worker or other mental healthcare provider (19)
- Other type of health worker that deals directly with patients. Please specify (18) ________________________________________________

Display This Question:

If What is your profession? = Doctor (MD, DO)

Q2.9 Select the level of your profession:

- Intern (1)
- Resident (2)
- Fellow (3)
- Attending (4)

Display This Question:

If What is your profession? = Doctor (MD, DO)

Q2.10 What best describes your speciality?

- Critical Care/Pulmonology (4)
- Emergency Medicine (5)
- Internal Medicine Hospitalist (6)
- Outpatient General Internist (7)
- Family Medicine (8)
- Pediatrics (9)
- Obstetrics/Gynecology (15)
- Infectious Disease (10)
- Anesthesiology (11)
- Psychiatry/Psychology (16)
- Medicine SubSpecialty (Describe): (13) ________________________________________________
- Surgical Specialty Physician (Describe): (14) ________________________________________________
- Other: (12) ________________________________________________

Q2.11 In which type of health care facility do you primarily provide care for patients?

- Academic medical center (4)
- Government Health System (i.e. county or city hospital) (5)
- Community, private health system (6)
- VA health system (9)
- Prison or other detention health system (10)
- Long term care or assisted living (11)
- Out of hospital, ambulance etc. (12)
- Other: (8) ________________________________________________

Q2.12 Do you have previous experience in global health, military, and/or frontline health work in disasters?

- Yes (1)
- No (2)

Display This Question:

If Do you have previous experience in global health, military, and/or frontline health work in disas... = Yes

Q2.13 Which of the following reflect your prior experiences? Select all that apply.

- Global Health development work (1)
- Global Health in humanitarian settings (conflict or natural disasters) (2)
- Military (3)
- Disasters or emergencies in your home country (4)

Q2.14 Do you currently live with individuals who are elderly, have a chronic illness, or who for other reasons are at high risk for complications from COVID-19?

- Elderly (4)
- Chronic illness (6)
- Other high risk individuals (5)
- No (2)

Q2.15 Do you have any personal risk factors for COVID-19? If so, which of the following?

- Underlying medical condition (1)
- Immunocompromised (5)
- Pregnancy (6)
- Other (7)
- None (2)

| Page Break |  |
| --- | --- |

Q2.16 Your Work During the COVID-19 Pandemic:

Q2.17
Have you provided any clinical care (in-person or tele) for patients with or suspected of having COVID-19?

- Yes (1)
- No (2)

Display This Question:

If Have you provided any clinical care (in-person or tele) for patients with or suspected of having... = Yes

Q2.18 Please specify how you have provided care: Select all that apply.

- Telehealth (1)
- In-Person (3)
- Other: (2) ________________________________________________

Display This Question:

If Please specify how you have provided care: Select all that apply. = In-Person

Q2.19 In what setting(s) did you provide direct clinical care for patients with COVID-19 this year? Select all that apply.

- Emergency Department (3)
- Urgent Care (4)
- Outpatient Primary Care (1)
- Outpatient Speciality (2)
- Federally Qualified Health Center (9)
- Inpatient Non-Intensive or Non-Critical Care (5)
- Inpatient Intensive or Critical Care (6)
- Specialized COVID unit/tent (7)
- Home Health Care Service (10)
- Long term care or assisted living (11)
- Other: (8) ________________________________________________

Display This Question:

If Have you provided any clinical care (in-person or tele) for patients with or suspected of having... = Yes

Q2.20 The patient volume in my own clinical practice in the past 30 days has been:

- Much lower than usual for this time of year (4)
- Lower than usual for this time of year (5)
- The same usual for this time of year (7)
- More than usual for this time of year (8)
- Much more than usual for this time of year (10)

| Page Break |  |
| --- | --- |

End of Block: SECTION 2: Demographic Questions

Start of Block: SECTION 3: Personal Risks and Challenges

Q3.1 SECTION 3: Personal Risks and Challenges

Q3.2
Please rate from "Strongly Disagree" to "Strongly Agree" (1-5) your level of agreement with the following statements:

|  | Strongly Disagree (1) | Disagree (2) | Neither agree nor disagree (4) | Agree (6) | Strongly agree (7) | Not Applicable (9) | Prefer not to answer (10) |
| --- | --- | --- | --- | --- | --- | --- | --- |
| I feel worried about my personal health if/when providing direct in-person care to COVID-19 patients. (1) |  |  |  |  |  |  |  |
| After providing direct in-person care to COVID-19 patients I am worried about spreading COVID-19 to my family or friends. (2) |  |  |  |  |  |  |  |
| It is my professional duty to provide in-person care to patients with COVID-19 even if I cannot be provided with adequate personal protective equipment (PPE). (3) |  |  |  |  |  |  |  |
| I feel confident in my hospital or clinic leadership's actions to protect staff from risks from COVID-19. (5) |  |  |  |  |  |  |  |
| My health care facility has a solid plan for resuming non-COVID-related health care services that will continue to prioritize safety of healthcare workers and patients. (19) |  |  |  |  |  |  |  |

| Page Break |  |
| --- | --- |

Q3.3 My place of work has faced or is facing shortages of personal equipment (PPE).

- Yes (1)
- No (2)
- Unsure (3)

Q3.4
Please rate from "Strongly Disagree" to "Strongly Agree" (1-5) your level of agreement with the following statements:

|  | Strongly Disagree (1) | Disagree (2) | Neither agree nor disagree (4) | Agree (6) | Strongly agree (7) | Not Applicable (9) | Prefer not to answer (10) |
| --- | --- | --- | --- | --- | --- | --- | --- |
| My place of work has faced or is facing shortages of personal equipment (PPE). = No  Or My place of work has faced or is facing shortages of personal equipment (PPE). = Unsure  If my hospital or clinic runs out of adequate PPE, I will be unwilling to provide in-person to patients with COVID-19. (32) |  |  |  |  |  |  |  |
| I feel comfortable communicating with administration about safety issues in my institution related to the care of COVID-19 patients. If disagree/strongly disagree, please specify reason: (22) |  |  |  |  |  |  |  |
| I feel confident that if I spoke out publicly about safety issues in my institution related to the care of COVID-19 patients, I would not experience retaliation from my institution. If disagree/strongly disagree, please specify reason: (26) |  |  |  |  |  |  |  |
| I am willing to do tasks outside my own formal training to care for critically ill patients with COVID-19. (31) |  |  |  |  |  |  |  |

| Page Break |  |
| --- | --- |

End of Block: SECTION 3: Personal Risks and Challenges

Start of Block: Section 4: Ethical Decision Making and Resource Allocation

Q4.1 SECTION 4: Ethical Decision Making and Resource Allocation
 
 The COVID-19 pandemic has the potential to overwhelm healthcare systems and create situations in which there are not enough lifesaving resources such as hospital beds and ventilators for all the patients who need them. The following section will ask about situations with limited resources and the distress healthcare providers may face in this situation.

Q4.2
Please rate from "Strongly Disagree" to "Strongly Agree" (1-5) your level of agreement with the following statements with respect to the COVID-19 pandemic:

|  | Strongly disagree (11) | Disagree (12) | Neither agree nor disagree (14) | Agree (16) | Strongly agree (17) | Not Applicable (18) | Prefer Not to Answer (19) |
| --- | --- | --- | --- | --- | --- | --- | --- |
| Before COVID-19, I was trained and/or had significant experience with priority setting with limited resources. (1) |  |  |  |  |  |  |  |
| I have received sufficient training and preparation in how to allocate scarce resources to patients amidst the current COVID-19 pandemic. (2) |  |  |  |  |  |  |  |
| My health care facility has given me clear information about how scarce resources will be allocated here if necessary so the burden of making decisions will not fall on the bedside team. (3) |  |  |  |  |  |  |  |
| I am worried I will be required to personally make decisions about allocating limited resources, like which patients get ventilators or other life-saving resources, in the moment based on my own judgment. (6) |  |  |  |  |  |  |  |

| Page Break |  |
| --- | --- |

Q4.3 I am worried about feeling stress when allocating limited resources due to conflict between institutional constraints or procedures and what I believe is right.

- Yes, right now (1)
- Yes, in the future (2)
- No (3)

Q4.4 My facility or office is actively limiting/rationing (Select all that apply):

- Personal protective equipment (PPE) (6)
- Disinfectants, sanitizers and other cleaning supplies (9)
- Diagnostic testing (such as COVID-19 tests or antibody tests) (1)
- Ventilators/Respirators (2)
- Other modes of assisted ventilation (such as BiPAP and CPAP) (8)
- Other therapeutic equipment (3)
- Hospital beds (4)
- None of the above (5)

| Page Break |  |
| --- | --- |

| 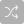 |
| --- |

Q4.5 Imagine you were required to make your institution’s policy on how to ration scarce life-saving resources to patients. Of the following SEVEN approaches, please rank in order how you might prioritize patients. Please mark 1 for your first choice, 2 for your second choice, 3 for your third choice, etc. For each, please feel free to write notes or comments.

______ Prioritize people who are important political, business, or cultural figures. (1)

______ Prioritize healthcare workers. (9)

______ Prioritize young people who have greater potential to live a longer life. (3)

______ Prioritize people who are most likely to survive based on clinical picture regardless of other factors. (4)

______ Prioritize the sickest people regardless of other factors. (5)

______ Use a "First Come, First Served" approach until equipment runs out. (6)

______ Use a lottery system to give everyone a fair shot. (7)

______ Another prioritization approach. (10)

| Page Break |  |
| --- | --- |

End of Block: Section 4: Ethical Decision Making and Resource Allocation

Start of Block: SECTION 5: Additional Information

Q5.1
SECTION 5: Additional Information

Q5.2
Thank you for taking the time to complete this survey. This information is critical to inform how clinicians respond to the COVID-19 crisis and ensure that the voices of health workers are heard in policy making around protection and ethical decision making.


In the space below, leave any additional thoughts or clarifications regarding the questions asked in this survey.

________________________________________________________________

________________________________________________________________

________________________________________________________________

________________________________________________________________

________________________________________________________________

End of Block: SECTION 5: Additional Information
